# Association of Body Mass Index With Somatic Mutations in Breast Cancer

**DOI:** 10.3389/fonc.2021.613933

**Published:** 2021-04-01

**Authors:** Bo Chen, Liping Guo, Kai Li, Weikai Xiao, Yingzi Li, Cheukfai Li, Hsiaopei Mok, Li Cao, Jiali Lin, Guangnan Wei, Guochun Zhang, Ning Liao

**Affiliations:** ^1^ Department of Breast Cancer, Cancer Center, Guangdong Provincial People’s Hospital and Guangdong Academy of Medical Sciences, Guangzhou, China; ^2^ The Second School of Clinical Medicine, Southern Medical University, Guangzhou, China; ^3^ School of Medicine, South China University of Technology, Guangzhou, China

**Keywords:** breast cancer, body mass index, somatic mutations, genomic, next-generation sequencing

## Abstract

**Background:**

The relationship between body mass index (BMI) and the prognosis or treatment response in patients with breast cancer has been demonstrated in previous studies, but the somatic mutation profiles in breast cancer patients with different BMIs have not been explored.

**Methods:**

In the present study, the somatic mutation profiles in 421 female breast cancer patients who were stratified into three subgroups based on BMI (normal weight, overweight/obese, and underweight) were investigated. Capture-based targeted sequencing was performed using a panel comprising 520 cancer-related genes.

**Results:**

A total of 3547 mutations were detected in 390 genes. In breast cancer patients with different BMI statuses, the tumors exhibited high mutation frequency and burden. *TP53* was the most common gene in the three groups, followed by *PIK3CA*, *ERBB2*, and *CDK12.* Meanwhile, the mutation hotspots in *TP53* and *PIK3CA* were the same in the three BMI groups. More *JAK1* mutations were identified in underweight patients than those in normal patients. Except for *JAK1*, differentially mutated genes in postmenopausal patients were completely different from those in premenopausal patients. The distribution of mutation types was significantly different among BMI groups in the postmenopausal group. Underweight patients in the postmenopausal group harbored more *TP53* mutations, more amplifications, and more mutations in genes involved in the WNT signaling pathway.

**Conclusions:**

Our next-generation sequencing (NGS)-based gene panel analysis revealed the gene expression profiles of breast cancer patients with different BMI statuses. Although genes with high mutation frequency and burden were found in different BMI groups, some subtle differences could not be ignored. *JAK1* mutations might play a vital role in the progression of breast cancer in underweight patients, and this needs further analysis. Postmenopausal underweight patients with breast cancer have more aggressive characteristics, such as *TP53* mutations, more amplifications, and more mutations in genes involved in the WNT signaling pathway. This study provides new evidence for understanding the characteristics of breast cancer patients with different BMIs.

## Background

Breast cancer imposes high healthcare burden worldwide (1). It has a complex etiology, involving genetic variants, environmental and behavioral factors, and their interactions (2, 3). The body mass index (BMI, calculated as weight in kilograms divided by height in meters squared) is widely used to classify body size as normal, underweight, overweight, or obese. Many studies have investigated the association of weight or weight change with prognosis among patients with breast cancer (4). Observational studies revealed the association between overweight/obesity and several measures of reduced prognosis in patients with breast cancer, and some instances of lower survival in women who are underweight or who experience unexplained weight loss after diagnosis. Our previous study demonstrated that being underweight was an independent prognostic factor for poor overall survival in young breast cancer patients with axillary lymph node metastasis or stage III-IV (5). A recent study found that overweight/obese patients with breast cancer treated with a docetaxel-based adjuvant chemotherapy regimen presented worse prognosis and an increased risk of developing distant metastases than lean patients with breast cancer treated with the same chemotherapy regimen (6). A systematic review revealed a negative effect of obesity on aromatase inhibitor efficacy in postmenopausal patients with hormone receptor-positive breast cancer (7).

Several mechanisms have been proposed to explain the relationship between breast cancer prognosis/treatment response and BMI. These mechanisms include increased tissue or circulating levels of metabolic and sex hormones, increased levels of inflammation and other adipocytokines, and decreased levels of hormone-binding proteins in obese patients. In recent decades, high-throughput technology has significantly accelerated our understanding of the molecular pathways underlying breast cancer (8). However, the association between BMI and genomic alterations in breast cancer remains largely unexplored.

In the present study, we investigated the somatic mutation profiles in female patients with breast cancer who were stratified into three subgroups based on BMI (normal weight, overweight/obese, and underweight). Our aim was to identify different gene expression patterns in breast cancer patients with different BMIs. Investigation of the mechanism underlying this association may provide insights into the phenomenon of breast cancer progression and might lead to the development of better prevention efforts and treatment.

## Materials and Methods

### Patients and Specimens

In this study, we enrolled 421 female breast cancer patients from the Guangdong Provincial People’s Hospital (GDPH), from June 1, 2017 to September 27, 2018. The clinical data were obtained from the electronic medical records. The American Society of Clinical Oncology (ASCO)/College of American Pathologists (CAP) guidelines were used to define estrogen receptor (ER)-, progesterone receptor (PR)-, and human epidermal growth factor receptor 2 (HER2)-positivity. Breast cancer tissue samples were obtained through biopsy or surgery and processed into formalin-fixed, paraffin-embedded (FFPE) cell blocks. All the samples were evaluated by two pathologists. The height and weight of the patient before treatment were used in calculating the baseline BMI. Baseline BMI and qualified primary tumor tissue sequencing information was available for all patients. This study was reviewed and approved by the Ethic Guangdong Provincial People’s Hospital (No. GDREC2014122H). The collection and use of tissues followed procedures that are in accordance with the ethical standards formulated in the Declaration of Helsinki. All patients provided written informed consent before study entry.

### Tissue DNA Extraction, Next-Generation Sequencing (NGS) Library Preparation, and Capture-Based Targeted DNA Sequencing

This investigation was conducted in accordance with our previous study (9, 10). DNA was extracted using the QIAamp DNA FFPE tissue kit (Qiagen, Hilden, Germany), and DNA concentration was measured using the Qubit dsDNA assay. Genomic profiling was performed using a panel covering 520 cancer-related genes (OncoScreen Plus, Burning Rock Biotech, Guangzhou, China). Events were then classified as germline or somatic depending on their presence in the matched normal set (white blood cells) of events. All data can be viewed in NODE (http://www.biosino.org/node) by pasting the accession (OEP001295) into the text search box or through the URL: http://www.biosino.org/node/project/detail/OEP001295.

### Sequence Data Analysis

Sequencing assays were performed, while being blinded to the clinical pathological parameters, at Burning Rock Biotech, a CLIA-certified company in Guangzhou, China.

### Clinicopathological Characteristics

Of the 421 breast cancer patients recruited, 37, 284, and 100 patients had BMIs of <18.5 kg/m^2^ (underweight, UW group), 18.5 to 24.9 kg/m^2^ (normal weight, NW group), and ≥ 25.0 kg/m^2^ (overweight/obese, OW group), respectively. The median age at the time of diagnosis was different among UW (44 years; range, 27 to 74 years), NW (47 years; range, 22 to 85 years), and OW patients (51 years; range, 32 to 79 years). Moreover, the BMI classification revealed a significant association with age at onset (*P* = 0.022) and menopausal status (*P* = 0.018). There were no significant correlations between BMI classification and other clinicopathologic factors, these are listed in **Table 1**. All patients received standard-of-care treatment according to the National Comprehensive Cancer Network guidelines.

**Table 1:** Clinicopathological characteristics among three groups.

Characteristics	UW (n = 37)		NW (n = 284)		OW (n = 100)		P-value
**Age**							0.022*
Median (range)	44 (27~74)		47 (22~85)		51 (32~79)		
<=40 years	12	32.43%	66	23.24%	13	13.00%	
>40 years	25	67.57%	218	76.76%	87	87.00%	
**Menopausal status**							0.018*
Pre	26	70.27%	172	60.56%	47	47.00%	
Post	11	29.73%	112	39.44%	53	53.00%	
**T stage**							0.131
T1	11	29.73%	107	37.68%	28	28.00%	
T2	24	64.86%	149	52.46%	62	62.00%	
T3	2	5.41%	14	4.93%	9	9.00%	
T4	0	0.00%	13	4.58%	1	1.00%	
Unknown	0	0.00%	1	0.35%	0	0.00%	
**N stage**							0.948
N0	14	37.84%	118	41.55%	44	44.00%	
N1	14	37.84%	94	33.10%	33	33.00%	
N2	8	21.62%	53	18.66%	17	17.00%	
N3	1	2.70%	19	6.69%	6	6.00%	
**M stage**							1
M0	35	94.59%	270	95.07%	94	94.00%	
M1	2	5.41%	14	4.93%	5	5.00%	
Unknown	0	0.00%	0	0.00%	1	1.00%	
**Pathologic stage**							0.878
IA	7	18.92%	67	23.59%	14	14.00%	
IIA	10	27.03%	77	27.11%	31	31.00%	
IIB	11	29.73%	60	21.13%	26	26.00%	
IIIA	6	16.22%	44	15.49%	15	15.00%	
IIIB	0	0.00%	5	1.76%	2	2.00%	
IIIC	1	2.70%	17	5.99%	6	6.00%	
IV	2	5.41%	14	4.93%	5	5.00%	
Unknown	0	0.00%	0	0.00%	1	1.00%	
**Histological grade**							0.285
I	2	5.41%	8	2.82%	3	3.00%	
II	13	35.14%	124	43.66%	53	53.00%	
III	21	56.76%	144	50.70%	43	43.00%	
Unknown	1	2.70%	8	2.82%	1	1.00%	
**Histological type**							0.422
DCIS	2	5.41%	3	1.06%	1	1.00%	
Infiltrating ductal Carcinoma	32	86.49%	249	87.68%	90	90.00%	
Infiltrating lobular Carcinoma	0	0.00%	11	3.87%	4	4.00%	
Others	3	8.11%	21	7.39%	5	5.00%	
**ER status**							1
Negative	10	27.03%	80	28.17%	28	28.00%	
Positive	27	72.97%	204	71.83%	72	72.00%	
**PR status**							0.711
Negative	12	32.43%	98	34.51%	30	30.00%	
Positive	25	67.57%	186	65.49%	70	70.00%	
**HR status**							0.919
Negative	9	24.32%	72	25.35%	23	23.00%	
Positive	28	75.68%	212	74.65%	77	77.00%	
**HER2 status**							0.65
Negative	22	59.46%	185	65.14%	65	65.00%	
Positive	14	37.84%	86	30.28%	28	28.00%	
Equivocal	1	2.70%	12	4.23%	7	7.00%	
Unknown	0	0.00%	1	0.35%	0	0.00%	
**Ki67 status**							0.269
<14	5	13.51%	69	24.30%	19	19.00%	
>=14	32	86.49%	214	75.35%	80	80.00%	
Unknown	0	0.00%	1	0.35%	1	1.00%	

P-value: Using Fisher’s exact test, “Unknown” ignored. (N stage, Pathologic stage: chisq.test).

*P < 0.05 was statistically significant.

### Statistical Analyses

Data were summarized by frequency and percentage for categorical variables including mutation detection rate (variation rate) and distribution of mutation types. We ranked these genes in each group by their variation rate. A common alterations gene was defined as the genes of the top 10 variation rate in each group. Demographic, clinical, and pathologic characteristics were compared using the Chi-squared test or Fisher’s exact test (categorical variables), as applicable. The differences were considered significant at *P *< 0.05. Data were analyzed using the SPSS 23.0 software (Chicago, IL, USA).

## Results

### Mutation Landscape of Breast Tumors in Different BMI Groups

**Figure 1:**
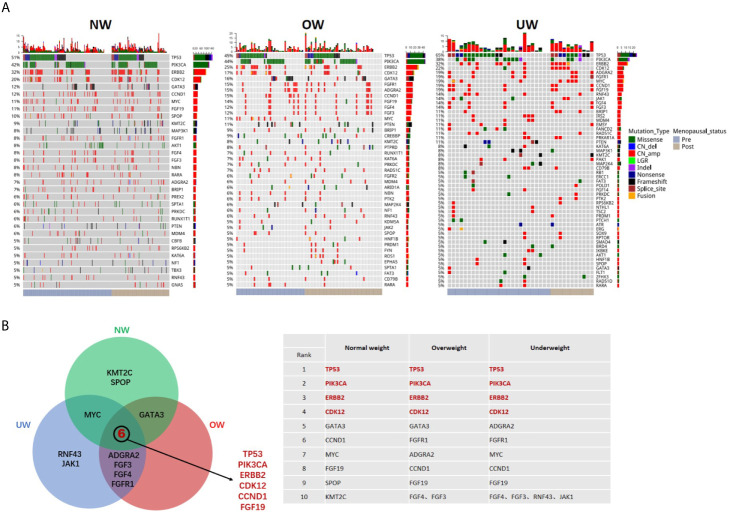
Mutation landscape of breast tumors in different BMI groups **(A)** Summary of the genomic features of the 421 breast cancer patients. Left, normal weight group (NW); middle, overweight group (OW); right, underweight group (UW). Genomic alterations of more than 5% are shown in the Oncoprint. **(B)** Common mutated genes (top ten in each group) between the three groups were identified using a Venn diagram.

A total of 3547 mutations, including 1765 single nucleotide variants (SNVs), 1645 copy number (CN) amplifications, 54 insertions or deletions (Indels), 63 fusions, 15 deletions, and 5 large genomic rearrangements (LGR) were detected in 390 genes. The mutation landscape of the three groups is depicted in **Figure 1A**. Common mutation overlap between the three groups was identified using a Venn diagram (**Figure 1B**). The results demonstrated that the top mutated genes were common among the three groups. *TP53* was the most common gene in the three groups, followed by *PIK3CA*, *ERBB2*, and *CDK12.*

### The Spectrum of *TP53* and *PIK3CA* Mutations

**Figure 2:**
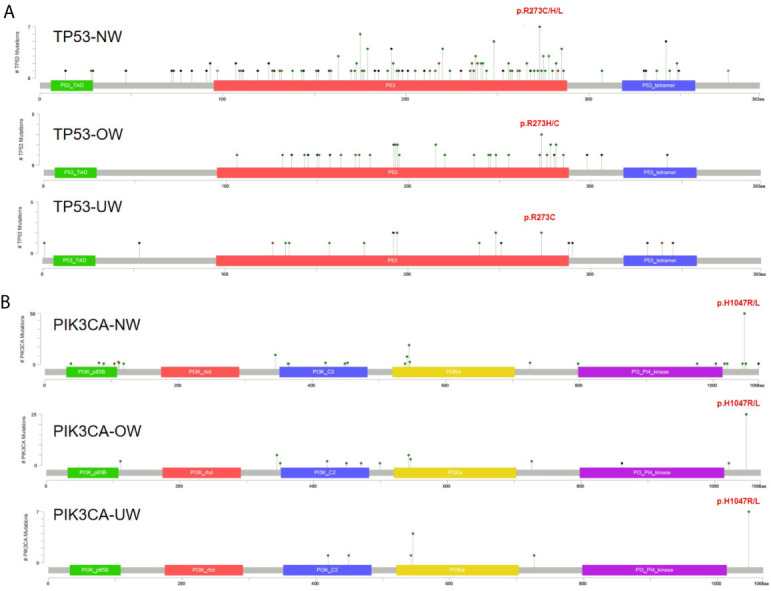
The spectrum of *TP53* and *PIK3CA* mutations **(A)** *TP53* mutations in different BMI groups. **(B)** *PIK3CA* mutations in different BMI groups.

As *TP53* and *PIK3CA* were the most frequently mutated genes, we further investigated the *TP53* and *PIK3CA* mutation spectrum in different BMI groups. **Figure 2A** illustrates that the distribution of *TP53* mutations was similar among the three groups. A hotspot in codon 273 was detected in the NW (R273C/H/L, n = 7, n is the number of mutations) and OW groups (R273H/C, n = 3), while two mutations in codon 273 were also observed in the UW group. We identified the mutation in codon 1047 (H1047R/L) of *PIK3CA* to be the most frequent in the three groups (n = 58 in NW group; n = 25 in OW group; n = 7 in NW group; **Figure 2B**).

### Identification of Differentially Mutated Genes

**Figure 3:**
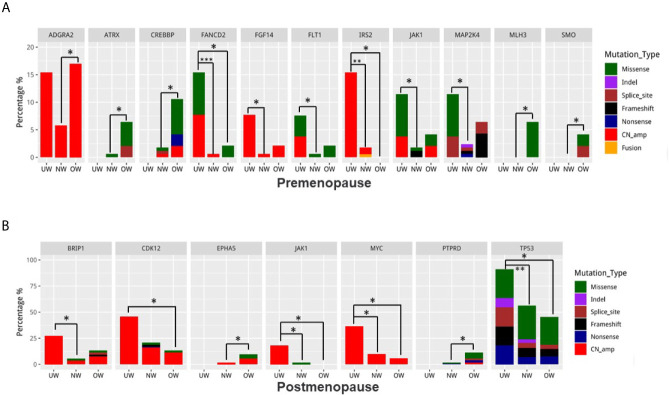
Identification of differentially mutated genes Differences in mutation frequencies among the normal weight, overweight, and underweight groups in **(A)** premenopausal patients and **(B)** postmenopausal patients. For each gene, the left bar represents the underweight group, the middle bar represents the normal weight group, and the right bar represents the overweight group. *P < 0.05, **P < 0.01, ***P < 0.001.

Next, we compared the differentially mutated genes in the different BMI groups. After patient stratification based on menstrual status, we found that the identity of differentially mutated genes in premenopausal patients (UW: 26 patients; NW: 172 patients; OW: 47 patients) with breast cancer differed from that in postmenopausal patients (UW: 11 patients; NW: 112 patients; OW: 53 patients). In premenopausal patients (**Figure 3A**), underweight patients carried more *FANCD2*, *FGF14*, *FLT1*, *IRS2*, *JAK1*, and *MAP2K4* variants. The mutation frequencies of *ATRX* and *CREBBP* were significantly elevated in overweight/obese patients. Notably, mutations in *MLH3* (n = 3) and *SMO* (n = 2) were exclusively detected in overweight patients. Moreover, UW and OW groups had a higher *ADGRA2* amplification rate than the NW group (UW vs NW vs OW = 19.4% vs 7.6% vs 15.3%, NW vs UW: *P* = 0.030; NW vs OW: *P* = 0.044). Except for *JAK1*, differentially mutated genes in postmenopausal patients were completely different from those in premenopausal patients (**Figure 3B**). In postmenopausal patients with breast cancer, we found that underweight patients harbored significantly more *BRIP1*, *CDK12*, *JAK1*, *MYC*, and *TP53* mutations, whereas overweight patients harbored more *EPHA5* and *PTPRD* mutations. The *P*-values of identification are listed in **Supplementary Table S1**.

### Mutation-Type Distribution and Pathway Analysis

**Figure 4:**
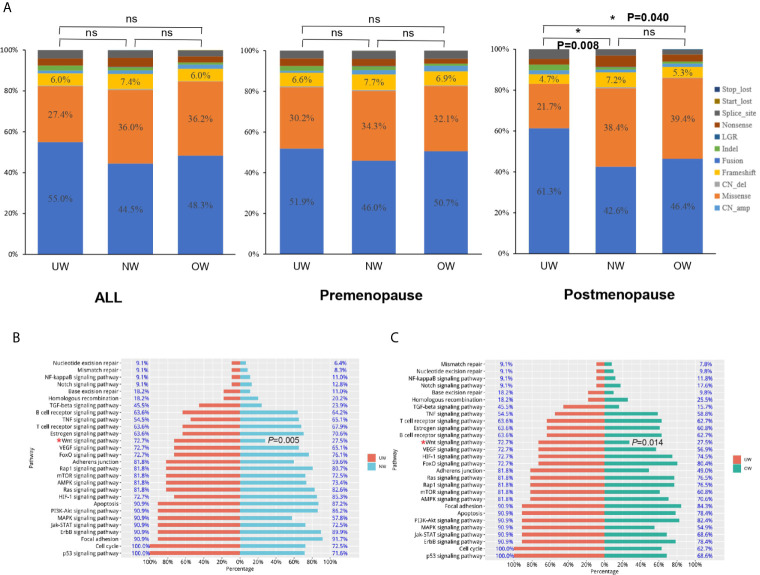
Mutation-type distribution and pathway analysis **(A)** The mutation-type distribution of different BMI groups. In postmenopausal patients, the underweight group harbored significantly more mutations in genes involved in the WNT signaling pathway than those in genes in the **(B)** normal weight and **(C)** overweight groups (UW vs NW: *P* = 0.005 and UW vs OW: *P* = 0.014). *P < 0.05; NS *P* ≤ 0.05.

As shown in **Figure 4A**, the distribution of mutation types was not significantly different among different BMI groups in all or premenopausal patients (all: P-value>0.05; premenopausal: P-value>0.05). In postmenopausal patients, amplification percentage was larger and the percentage of missense mutations was lower in underweight patients than those in normal or overweight patients (UW vs NW vs OW, amplification: 61.32% vs 42.59% vs 46.41%, missense mutations: 21.70% vs 38.37% vs 39.43%, NW vs UW: *P* = 0.008 and OW vs UW: *P* = 0.040). Next, we used the Kyoto Encyclopedia of Genes and Genomes (KEGG) database to analyze the enriched pathways in the three groups. KEGG pathway analysis revealed that underweight patients harbored significantly more mutations in genes involved in the WNT signaling pathway in the postmenopausal group (UW vs NW vs OW, 72.7% vs. 27.5% 27.5%, UW vs NW: *P* = 0.005 and UW vs OW: *P* = 0.014) (**Figures 4B, C**). However, pathway analysis did not reveal any significant difference among the different BMI groups (overall or in premenopausal patients).

### Tumor Mutation Burden Was Similar Among the Different BMI Groups

**Figure 5:**
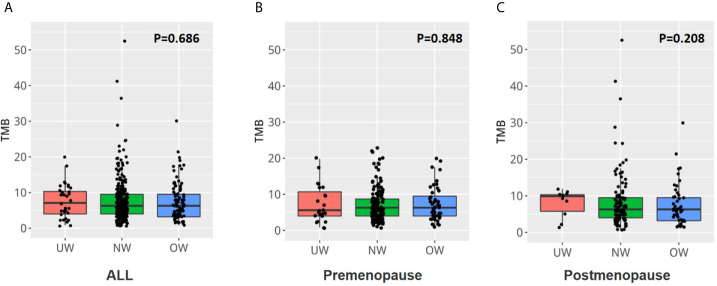
The tumor mutation burden was similar among the different BMI groups **(A)** The tumor mutation burden was similar in the three groups (*P* = 0.686). The tumor mutation burden was also comparable among the different BMI groups in **(B)** premenopausal or **(C)** postmenopausal patients with breast cancer.

The respective median and mean mutation burden of tumors in the overall population were 7.1 and 7.5 (range, 0.8 to 19.8) in the UW group, 6.3 and 7.7 (range, 0.8 to 52.4) in the NW group, and 6.3 and 7.3 (range, 0.8 to 30.2) in the OW group. No significant difference was identified between the three groups (*P* = 0.686) (**Figure 5A** and **Supplementary Table S2**). Additionally, the tumor mutation burden was also comparable among the different BMI groups in premenopausal (**Figure 5B**, *P* = 0.848) or postmenopausal patients with breast cancer (**Figure 5C**, *P* = 0.208).

## Discussion

In recent decades, the impact of obesity or being underweight on cancer progression has attracted much attention (2). However, little is known about the influence of BMI on gene expression profiles in breast cancer. In this study, we performed NGS on tumor tissues to explore the somatic mutation profiles in 421 female breast cancer patients with different BMI statuses.

Genomic aberrations contribute to breast cancer initiation and progression (11). In this study, we found that the tumors in breast cancer patients with different BMI statuses exhibit high mutation frequency and burden. However, there are certain differences in the age composition among these three patient groups. Consistent with the overwhelming evidence from many studies (12, 13), we found *TP53* and *PIK3CA* to be the most common genes playing a very important role in the progression of breast cancer (14, 15). Meanwhile, the three BMI groups harbored the same *TP53* and *PIK3CA* mutation hotspots. Therefore, we believe that the tumor-driver genes in these three patient groups are the same.

The assumption of a causal relation between excess body weight and DNA damage is strongly supported by mechanistic studies (16). The formation of reactive oxygen species may be the consequence of increased insulin and glucose levels and may induce oxidative DNA damage either directly, or *via* the formation of lipid peroxidation products. Failure to properly repair DNA damage may result in cell death or genomic instability which may eventually lead to cancer (17, 18). Therefore, we assumed that some different tumor mutations might be harbored by breast cancer patients with different BMIs. With respect to these tumor mutations, we found some subtle differences among breast cancer patients with different BMIs; for example, more *JAK1* mutations were found in underweight patients. *JAK1* is a member of the Janus kinase family of proteins and is essential for IL-6‒mediated inflammatory signaling (19). It plays a critical role in the progression of metastatic cancer (20). In a previous study, we found that the levels of *JAK1* mRNA were correlated with prognosis and immune infiltration in breast cancer (21). Whether *JAK1* mutations can induce changes in the tumor immune microenvironment in underweight patients requires further study.

Previous studies have indicated that under different menstrual states, BMI affects the prognosis of breast cancer patients differently. Therefore, menopausal status was an important stratification factor in this study. Except for *JAK1*, genes differentially mutated in postmenopausal patients were completely different from those in premenopausal patients. The distribution of mutation types was significantly different between the different BMI groups in the postmenopausal group. Underweight patients in the postmenopausal group harbored more *TP53* mutations, more amplifications, and more mutations in genes involved in the WNT signaling pathway. A higher *TP53* mutation rate has been reported in breast cancer patients with aggressive characteristics (22, 23). DNA amplification is a ubiquitous mechanism of oncogene activation in cancers (24). The amplification and overexpression of the identified genes provide tumor cells with selective advantages and lead to unlimited cell growth. In addition, gene amplification is a common form of genomic instability (25). The activation of the WNT signaling pathway contributes to tumor recurrence. The WNT pathway crosstalks with the Notch and Sonic hedgehog pathways, and this crosstalk can be targeted for treating various cancers (26). Therefore, the tumor mutation characteristics of underweight patients after menopause might indicate that their tumors were more malignant and heterogenous.

Our study has several limitations. First, all the enrolled patients were Chinese. The mutational landscape and genomic signatures differ across ethnicities (27). Therefore, caution should be exercised when extrapolating these results to other ethnic groups. Second, our databases provide BMI values only at the time of the initial diagnosis. Third, we were unable to perform statistical analysis for investigating the survival and prognosis of these patients, because all the patients were followed-up for less than five years.

## Conclusions

In conclusion, our NGS-based gene panel analysis presents the gene expression profiles of breast cancer patients with different BMI statuses. Although genes with a high frequency of mutation and tumor mutation burden were identified in different BMI groups, some subtle differences could not be ignored. *JAK1* mutations might play a vital role in the progression of breast cancer in underweight patients, but further studies are needed before reaching a conclusion. Postmenopausal underweight patients with breast cancer and more aggressive characteristics, such as *TP53* mutations, more amplifications, and mutations in genes involved in the WNT signaling pathway were observed. This study provides new evidence for understanding the characteristics of breast cancer patients with different BMIs.

## Data Availability Statement

The datasets presented in this study can be found in online repositories. The names of the repository/repositories and accession number(s) can be found below: NODE [accession: OEP001295]; http://www.biosino.org/node/project/detail/OEP001295.

## Ethics Statement

The studies involving human participants were reviewed and approved by Department of Ethic Committee of Guangdong Provincial People’s Hospital. The patients/participants provided their written informed consent to participate in this study.

## Author Contributions

BC and NL designed and funded the study. BC, LG, KL, WX, CL, HM, and LC conducted the experiments and analyzed the data. GZ and NL provided supervision and suggestions for the implementation of the study. BC, LG, YL, JL, and GW drafted the manuscript. All authors contributed to the article and approved the submitted version.

## Funding

This work was supported by funds from the National Natural Science Foundation of China (81902828, BC); the High-level Hospital Construction Project (DFJH201921, BC); the Fundamental Research Funds for the Central Universities (y2syD2192230, BC); The Doctor Launch Fund of Guangdong Provincial People’s Hospital (2018bq02, BC); the Natural Science Foundation of Guangdong Province (2016A030313768, NL; 2018A030313292), the Research Funds from Guangzhou Municipal Science and Technology Project (201707010418, NL); and the Medical Scientific Research Foundation of Guangdong Province (B2019039, BC).

## Conflict of Interest

The authors declare that the research was conducted in the absence of any commercial or financial relationships that could be construed as a potential conflict of interest.

## Abbreviations

ASCO, American Society of Clinical Oncology; BMI, body mass index; CAP, College of American Pathologists; CN, copy number; ER, estrogen receptor; FFPE, formalin-fixed and paraffin-embedded; GDPH, Guangdong Provincial People’s Hospital; HER2, human epidermal growth factor receptor 2; Indels, insertions or deletions; KEGG, Kyoto Encyclopedia of Genes and Genomes; LGR, large genomic rearrangements; NW, normal weight; OW, overweight; PR, progesterone receptor; SNVs, single nucleotide variants; UW, underweight.
