# Supplementary material for: Association of Body Mass Index With Somatic Mutations in Breast Cancer
Source: Front Oncol. 2021 Apr 1;11:613933. doi: 10.3389/fonc.2021.613933 (PMC8049504; doi:10.3389/fonc.2021.613933)
Supplement: Supplementary file 1 [file Table_1.docx]

| **Gene** | **UW.Count** | **UW.Frequency** | **NW.Count** | **NW.Frequency** | **OW.Count** | **OW.Frequency** | **P-value**  **UW_vs_NW** | **P-value**  **UW_vs_OW** | **P-value**  **NW_vs_OW** | **Figure** |
| --- | --- | --- | --- | --- | --- | --- | --- | --- | --- | --- |
| **ADGRA2** | 4 | 16.0% | 10 | 6.0% | 8 | 17.0% | 0.092 | 1.000 | 0.032 | Figure 3A |
| **ATRX** | 0 | 0.0% | 1 | 0.6% | 3 | 6.4% | 1.000 | 0.547 | 0.034 | Figure 3A |
| **CREBBP** | 0 | 0.0% | 3 | 1.8% | 5 | 10.6% | 1.000 | 0.156 | 0.014 | Figure 3A |
| **FANCD2** | 4 | 16.0% | 1 | 0.6% | 1 | 2.1% | 0.001 | 0.046 | 0.393 | Figure 3A |
| **FGF14** | 2 | 8.0% | 1 | 0.6% | 1 | 2.1% | 0.046 | 0.275 | 0.393 | Figure 3A |
| **FLT1** | 2 | 8.0% | 1 | 0.6% | 1 | 2.1% | 0.046 | 0.275 | 0.393 | Figure 3A |
| **IRS2** | 4 | 16.0% | 3 | 1.8% | 0 | 0.0% | 0.006 | 0.012 | 1.000 | Figure 3A |
| **JAK1** | 3 | 12.0% | 3 | 1.8% | 2 | 4.3% | 0.031 | 0.334 | 0.305 | Figure 3A |
| **MAP2K4** | 3 | 12.0% | 4 | 2.4% | 3 | 6.4% | 0.049 | 0.412 | 0.182 | Figure 3A |
| **MLH3** | 0 | 0.0% | 0 | 0.0% | 3 | 6.4% | NA | 0.547 | 0.010 | Figure 3A |
| **SMO** | 0 | 0.0% | 0 | 0.0% | 2 | 4.3% | NA | 0.540 | 0.048 | Figure 3A |
| **BRIP1** | 3 | 27.3% | 6 | 5.5% | 7 | 13.7% | 0.036 | 0.363 | 0.117 | Figure 3B |
| **CDK12** | 5 | 45.5% | 23 | 21.1% | 7 | 13.7% | 0.126 | 0.029 | 0.385 | Figure 3B |
| **EPHA5** | 0 | 0.0% | 2 | 1.8% | 5 | 9.8% | 1.000 | 0.575 | 0.034 | Figure 3B |
| **JAK1** | 2 | 18.2% | 2 | 1.8% | 0 | 0.0% | 0.042 | 0.029 | 1.000 | Figure 3B |
| **MYC** | 4 | 36.4% | 11 | 10.1% | 3 | 5.9% | 0.031 | 0.015 | 0.551 | Figure 3B |
| **PTPRD** | 0 | 0.0% | 2 | 1.8% | 6 | 11.8% | 1.000 | 0.580 | 0.013 | Figure 3B |
| **TP53** | 10 | 90.9% | 63 | 57.8% | 24 | 47.1% | 0.049 | 0.009 | 0.235 | Figure 3B |

Supplementary Table S1. The *P*-value of identification of differentially mutation genes
