# Supplementary material for: Association of Body Mass Index With Somatic Mutations in Breast Cancer
Source: Front Oncol. 2021 Apr 1;11:613933. doi: 10.3389/fonc.2021.613933 (PMC8049504; doi:10.3389/fonc.2021.613933)
Supplement: Supplementary file 2 [file Table_2.docx]

| **Group** | **median** | **mean** | **Range (min~max)** | **Figure** |
| --- | --- | --- | --- | --- |
| UW | 7.1 | 7.5 | 0.8~19.8 | Figure 5A |
| NW | 6.3 | 7.7 | 0.8~52.4 | Figure 5A |
| OW | 6.3 | 7.3 | 0.8~30.2 | Figure 5A |
| UW | 5.6 | 7.3 | 0.8~19.8 | Figure 5B |
| NW | 6.3 | 7.1 | 0.8~23 | Figure 5B |
| OW | 6.3 | 7.3 | 0.8~19.8 | Figure 5B |
| UW | 9.9 | 8.1 | 1.6~11.9 | Figure 5C |
| NW | 6.3 | 8.6 | 0.8~52.4 | Figure 5C |
| OW | 6.3 | 7.4 | 1.6~30.2 | Figure 5C |

**Supplementary Table S2.** Tumor mutation burden in different BMI groups
