# Supplementary material for: Association of Body Mass Index With Somatic Mutations in Breast Cancer
Source: Front Oncol. 2021 Apr 1;11:613933. doi: 10.3389/fonc.2021.613933 (PMC8049504; doi:10.3389/fonc.2021.613933)
Supplement: Supplementary file 3 [file Table_3.docx]

**Supplementary Table S3.** The genes list of 520 cancer-related genes (OncoScreen Plus)

| **ABL1** NM_005157.5 | **CALR** NM_004343.3 | **DNMT1** NM_001130823.2 | **FGF6** NM_020996.2 | **HIST1H3J** NM_003535.2 |
| --- | --- | --- | --- | --- |
| **ABL2** NM_007314.3 | **CARD11** NM_032415.5 | **DNMT3A** NM_022552.4 | **FGF7** NM_002009.3 | **HIST2H3C** NM_021059.2 |
| **ACVR1** NM_001105.4 | **CASP8** NM_001228.4 | **DNMT3B** NM_006892.3 | **FGFR1** NM_023110.2 | **HIST2H3D** NM_001123375.2 |
| **ACVR1B** NM_020328.3 | **CBFB** NM_022845.2 | **DOT1L** NM_032482.2 | **FGFR2** NM_000141.4 | **HIST3H3** NM_003493.2 |
| **ADGRA2** NM_032777.9 | **CBL** NM_005188.3 | **E2F3** NM_001949.4 | **FGFR3** NM_000142.4 | **HLA-A** NM_001242758.1 |
| **AKT1** NM_001014432.1 | **CCND1** NM_053056.2 | **EED** NM_001308007.1 | **FGFR4** NM_002011.4 | **HNF1A** NM_000545.6 |
| **AKT2** NM_001626.5 | **CCND2** NM_001759.3 | **EGFL7** NM_016215.4 | **FH** NM_000143.3 | **HNF1B** NM_000458.3 |
| **AKT3** NM_005465.4 | **CCND3** NM_001760.4 | **EGFR** NM_005228.3 | **FLCN** NM_144997.5 | **HOXB13** NM_006361.5 |
| **ALK** NM_004304.4 | **CCNE1** NM_001238.3 | **EIF1AX** NM_001412.3 | **FLT1** NM_002019.4 | **HRAS** NM_005343.3 |
| **ALOX12B** NM_001139.2 | **CD274** NM_014143.3 | **EIF4A2** NM_001967.3 | **FLT3** NM_004119.2 | **HSD3B1** NM_000862.2 |
| **AMER1** NM_152424.3 | **CD276** NM_001024736.1 | **EIF4E** NM_001130679.1 | **FLT4** NM_182925.4 | **HSP90AA1** NM_001017963.2 |
| **ANKRD11** NM_001256182.1 | **CD79A** NM_001783.3 | **ELOC** NM_020341.3 | **FOXA1** NM_004496.3 | **ICOSLG** NM_001283050.1 |
| **APC** NM_000038.5 | **CD79B** NM_000626.3 | **EMSY** NM_001300942.1 | **FOXL2** NM_023067.3 | **ID3** NM_002167.4 |
| **APCDD1** NM_153000.4 | **CDC73** NM_024529.4 | **EP300** NM_001429.3 | **FOXO1** NM_002015.3 | **IDH1** NM_005896.3 |
| **AR** NM_000044.3 | **CDH1** NM_004360.4 | **EPCAM** NM_002354.2 | **FOXP1** NM_001244810.1 | **IDH2** NM_001289910.1 |
| **ARAF** NM_001256196.1 | **CDK12** NM_016507.3 | **EPHA2** NM_004431.4 | **FRS2** NM_001042555.2 | **IFNGR1** NM_000416.2 |
| **ARFRP1** NM_001267547.2 | **CDK4** NM_000075.3 | **EPHA3** NM_005233.5 | **FUBP1** NM_003902.4 | **IGF1** NM_001111285.2 |
| **ARID1A** NM_006015.4 | **CDK6** NM_001145306.1 | **EPHA5** NM_001281765.2 | **FYN** NM_002037.5 | **IGF1R** NM_000875.4 |
| **ARID1B** NM_020732.3 | **CDK8** NM_001260.2 | **EPHA7** NM_004440.3 | **GABRA6** NM_000811.2 | **IGF2** NM_000612.5 |
| **ARID2** NM_152641.2 | **CDKN1A** NM_001291549.1 | **EPHB1** NM_004441.4 | **GALNT12** NM_024642.4 | **IKBKE** NM_014002.3 |
| **ARID5B** NM_032199.2 | **CDKN1B** NM_004064.4 | **ERBB2** NM_004448.3 | **GATA1** NM_002049.3 | **IKZF1** NM_006060.5 |
| **ASXL1** NM_015338.5 | **CDKN1C** NM_000076.2 | **ERBB3** NM_001982.3 | **GATA2** NM_001145661.1 | **IL10** NM_000572.2 |
| **ASXL2** NM_018263.4 | **CDKN2A** NM_000077.4 | **ERBB4** NM_005235.2 | **GATA3** NM_001002295.1 | **IL7R** NM_002185.3 |
| **ATF1** NM_005171.4 | **CDKN2B** NM_004936.3 | **ERCC1** NM_202001.2 | **GATA4** NM_001308093.1 | **INHA** NM_002191.3 |
| **ATM** NM_000051.3 | **CDKN2C** NM_001262.2 | **ERCC2** NM_000400.3 | **GATA6** NM_005257.5 | **INHBA** NM_002192.3 |
| **ATR** NM_001184.3 | **CEBPA** NM_004364.4 | **ERCC3** NM_000122.1 | **GID4** NM_024052.4 | **INPP4A** NM_001134224.1 |
| **ATRX** NM_000489.4 | **CENPA** NM_001809.3 | **ERCC4** NM_005236.2 | **GLI1** NM_005269.2 | **INPP4B** NM_001101669.1 |
| **AURKA** NM_001323303.1 | **CHD1** NM_001270.2 | **ERCC5** NM_000123.3 | **GNA11** NM_002067.4 | **INSR** NM_000208.3 |
| **AURKB** NM_001284526.1 | **CHD2** NM_001271.3 | **ERG** NM_001136154.1 | **GNA13** NM_006572.5 | **IRF2** NM_002199.3 |
| **AXIN1** NM_003502.3 | **CHD4** NM_001273.3 | **ERRFI1** NM_018948.3 | **GNAQ** NM_002072.4 | **IRF4** NM_002460.3 |
| **AXIN2** NM_004655.3 | **CHEK1** NM_001274.5 | **ESR1** NM_000125.3 | **GNAS** NM_080425.3 | **IRS1** NM_005544.2 |
| **AXL** NM_021913.4 | **CHEK2** NM_007194.3 | **EWSR1** NM_013986.3 | **GPS2** NM_004489.4 | **IRS2** NM_003749.2 |
| **B2M** NM_004048.2 | **CHUK** NM_001278.4 | **EZH2** NM_004456.4 | **GREM1** NM_013372.6 | **JAK1** NM_001320923.1 |
| **BACH1** NM_001186.3 | **CIC** NM_015125.4 | **ABRAXAS1** NM_139076.2 | **GRIN2A** NM_000833.4 | **JAK2** NM_004972.3 |
| **BAP1** NM_004656.3 | **CRBN** NM_016302.3 | **TENT5C** NM_017709.3 | **GRM3** NM_000840.2 | **JAK3** NM_000215.3 |
| **BARD1** NM_000465.3 | **CREBBP** NM_004380.2 | **FANCA** NM_000135.2 | **GSK3B** NM_002093.3 | **JUN** NM_002228.3 |
| **BBC3** NM_001127240.2 | **CRKL** NM_005207.3 | **FANCC** NM_000136.2 | **GSTM1** NM_000561.3 | **KAT6A** NM_006766.4 |
| **BCL10** NM_003921.4 | **CRLF2** NM_022148.3 | **FANCD2** NM_001018115.2 | **GSTT1** NM_000853.3 | **KDM5A** NM_001042603.2 |
| **BCL2** NM_000633.2 | **CSF1R** NM_001288705.1 | **FANCE** NM_021922.2 | **H3F3A** NM_002107.4 | **KDM5C** NM_004187.3 |
| **BCL2L1** NM_001317919.1 | **CSF3R** NM_156039.3 | **FANCF** NM_022725.3 | **H3F3B** NM_005324.4 | **KDM6A** NM_001291415.1 |
| **BCL2L11** NM_001204107.1 | **CTCF** NM_006565.3 | **FANCG** NM_004629.1 | **H3F3C** NM_001013699.2 | **KDR** NM_002253.2 |
| **BCL2L2** NM_001199839.1 | **CTLA4** NM_005214.4 | **FANCI** NM_001113378.1 | **HDAC1** NM_004964.2 | **KEAP1** NM_012289.3 |
| **BCL6** NM_001130845.1 | **CTNNA1** NM_001323982.1 | **FANCL** NM_018062.3 | **HDAC2** NM_001527.3 | **KEL** NM_000420.2 |
| **BCOR** NM_001123383.1 | **CTNNB1** NM_001904.3 | **FANCM** NM_020937.3 | **HDAC4** NM_006037.3 | **KIT** NM_000222.2 |
| **BCORL1** NM_021946.4 | **CUL3** NM_001257198.1 | **FAS** NM_000043.5 | **HGF** NM_000601.5 | **KLF4** NM_001314052.1 |
| **BCR** NM_004327.3 | **CUL4A** NM_001008895.2 | **FAT1** NM_005245.3 | **HIST1H1C** NM_005319.3 | **KLHL6** NM_130446.2 |
| **BIRC3** NM_001165.4 | **CUL4B** NM_003588.3 | **FAT3** NM_001008781.2 | **HIST1H2BD** NM_021063.3 | **KMT2A** NM_001197104.1 |
| **BLM** NM_000057.3 | **CXCR4** NM_003467.2 | **FBXW7** NM_033632.3 | **HIST1H3A** NM_003529.2 | **KMT2C** NM_170606.2 |
| **BMPR1A** NM_004329.2 | **CYLD** NM_015247.2 | **FCGR2B** NM_004001.4 | **HIST1H3B** NM_003537.3 | **KMT2D** NM_003482.3 |
| **BRAF** NM_004333.4 | **CYP17A1** NM_000102.3 | **FGF10** NM_004465.1 | **HIST1H3C** NM_003531.2 | **KRAS** NM_033360.3 |
| **BRCA1** NM_007294.3 | **DAXX** NM_001141970.1 | **FGF12** NM_021032.4 | **HIST1H3D** NM_003530.4 | **LATS1** NM_004690.3 |
| **BRCA2** NM_000059.3 | **DCUN1D1** NM_020640.3 | **FGF14** NM_175929.2 | **HIST1H3E** NM_003532.2 | **LATS2** NM_014572.2 |
| **BRD4** NM_058243.2 | **DDR2** NM_001014796.1 | **FGF19** NM_005117.2 | **HIST1H3F** NM_021018.2 | **LMO1** NM_002315.2 |
| **BRIP1** NM_032043.2 | **DICER1** NM_177438.2 | **FGF23** NM_020638.2 | **HIST1H3G** NM_003534.2 | **LRP1B** NM_018557.2 |
| **BTG1** NM_001731.2 | **DIS3** NM_014953.4 | **FGF3** NM_005247.2 | **HIST1H3H** NM_003536.2 | **LYN** NM_002350.3 |
| **BTK** NM_000061.2 | **DNAJB1** NM_006145.2 | **FGF4** NM_002007.2 | **HIST1H3I** NM_003533.2 | **LZTR1** NM_006767.3 |
| **MAGI2** NM_012301.3 | **NOTCH1** NM_017617.4 | **PMS2** NM_000535.6 | **ROS1** NM_002944.2 | **TACC3** NM_006342.2 |
| **MALT1** NM_006785.3 | **NOTCH2** NM_024408.3 | **PNRC1** NM_006813.2 | **RPA1** NM_002945.3 | **TAF1** NM_001286074.1 |
| **MAP2K1** NM_002755.3 | **NOTCH3** NM_000435.2 | **POLD1** NM_001256849.1 | **RPS6KA4** NM_003942.2 | **TBX3** NM_016569.3 |
| **MAP2K2** NM_030662.3 | **NOTCH4** NM_004557.3 | **POLE** NM_006231.3 | **RPS6KB2** NM_003952.2 | **TCF3** NM_003200.3 |
| **MAP2K4** NM_001281435.1 | **NPM1** NM_002520.6 | **POM121L12** NM_182595.3 | **RPTOR** NM_020761.2 | **TCF7L2** NM_001146274.1 |
| **MAP3K1** NM_005921.1 | **NR4A3** NM_173200.2 | **PPM1D** NM_003620.3 | **RUNX1** NM_001754.4 | **TERC** NR_001566.1 |
| **MAP3K13** NM_001242314.1 | **NRAS** NM_002524.4 | **PPP2R1A** NM_014225.5 | **RUNX1T1** NM_001198679.1 | **TERT** NM_198253.2 |
| **MAP3K14** NM_003954.4 | **NRG1** NM_001322205.1 | **PPP2R2A** NM_133509.3 | **RYBP** NM_012234.6 | **TET1** NM_030625.2 |
| **MAPK1** NM_002745.4 | **NSD1** NM_022455.4 | **PPP6C** NM_001123355.1 | **SDHA** NM_004168.3 | **TET2** NM_001127208.2 |
| **MAPK3** NM_002746.2 | **NTHL1** NM_002528.6 | **PRDM1** NM_001198.3 | **SDHAF2** NM_017841.2 | **TGFBR1** NM_001306210.1 |
| **MAX** NM_002382.4 | **NTRK1** NM_001007792.1 | **PREX2** NM_024870.3 | **SDHB** NM_003000.2 | **TGFBR2** NM_001024847.2 |
| **MCL1** NM_021960.4 | **NTRK2** NM_006180.4 | **PRKAR1A** NM_002734.4 | **SDHC** NM_003001.3 | **TIPARP** NM_001184717.1 |
| **MDC1** NM_014641.2 | **NTRK3** NM_001012338.2 | **PRKCI** NM_002740.5 | **SDHD** NM_003002.3 | **TMEM127** NM_017849.3 |
| **MDM2** NM_002392.5 | **NUP93** NM_014669.4 | **PRKDC** NM_006904.6 | **SETD2** NM_014159.6 | **TMPRSS2** NM_001135099.1 |
| **MDM4** NM_002393.4 | **PAK1** NM_001128620.1 | **PRSS8** NM_002773.4 | **SF3B1** NM_012433.3 | **TNFAIP3** NM_001270507.1 |
| **MED12** NM_005120.2 | **PAK3** NM_001128168.2 | **PTCH1** NM_000264.3 | **SH2B3** NM_005475.2 | **TNFRSF14** NM_003820.3 |
| **MEF2B** NM_001145785.1 | **PAK5** NM_020341.3 | **PTEN** NM_000314.6 | **SH2D1A** NM_002351.4 | **TNFSF11** NM_003701.3 |
| **MEN1** NM_000244.3 | **PALB2** NM_024675.3 | **PTK2** NM_005607.4 | **SHQ1** NM_018130.2 | **TOP1** NM_003286.2 |
| **MET** NM_000245.3 | **PRKN** NM_004562.2 | **PTPN11** NM_002834.3 | **SLIT2** NM_004787.3 | **TOP2A** NM_001067.3 |
| **MGA** NM_001164273.1 | **PARP1** NM_001618.3 | **PTPRD** NM_002839.3 | **SLX4** NM_032444.2 | **TP53** NM_000546.5 |
| **MITF** NM_000248.3 | **PARP2** NM_005484.3 | **PTPRS** NM_002850.3 | **SMAD2** NM_001003652.3 | **TP63** NM_003722.4 |
| **MLH1** NM_000249.3 | **PARP3** NM_001003931.3 | **PTPRT** NM_133170.3 | **SMAD3** NM_005902.3 | **TRAF2** NM_021138.3 |
| **MLH3** NM_001040108.1 | **PARP4** NM_006437.3 | **QKI** NM_006775.2 | **SMAD4** NM_005359.5 | **TRAF7** NM_032271.2 |
| **MPL** NM_005373.2 | **PAX5** NM_016734.2 | **RAB35** NM_006861.6 | **SMARCA4** NM_001128849.1 | **TRRAP** NM_001244580.1 |
| **MRE11** NM_005591.3 | **PBRM1** NM_018313.4 | **RAC1** NM_018890.3 | **SMARCB1** NM_003073.4 | **TSC1** NM_000368.4 |
| **MSH2** NM_000251.2 | **PDCD1** NM_005018.2 | **RAD21** NM_006265.2 | **SMARCD1** NM_003076.4 | **TSC2** NM_000548.4 |
| **MSH3** NM_002439.4 | **PDCD1LG2** NM_025239.3 | **RAD50** NM_005732.3 | **SMO** NM_005631.4 | **TSHR** NM_000369.2 |
| **MSH6** NM_000179.2 | **PDGFRA** NM_006206.4 | **RAD51** NM_001164269.1 | **SNCAIP** NM_001308100.1 | **U2AF1** NM_001025203.1 |
| **MST1** NM_020998.3 | **PDGFRB** NM_002609.3 | **RAD51B** NM_133509.3 | **SOCS1** NM_003745.1 | **VEGFA** NM_001025366.2 |
| **MST1R** NM_002447.3 | **PDK1** NM_001278549.1 | **RAD51C** NM_058216.2 | **SOX10** NM_006941.3 | **VEGFB** NM_003377.4 |
| **MTOR** NM_004958.3 | **PDPK1** NM_002613.4 | **RAD51D** NM_002878.3 | **SOX17** NM_022454.3 | **VEGFC** NM_005429.4 |
| **MUTYH** NM_001128425.1 | **PGR** NM_000926.4 | **RAD52** NM_001297419.1 | **SOX2** NM_003106.3 | **VHL** NM_000551.3 |
| **MYC** NM_002467.4 | **PHOX2B** NM_003924.3 | **RAD54L** NM_003579.3 | **SOX9** NM_000346.3 | **VTCN1** NM_024626.3 |
| **MYCL** NM_001033082.2 | **PIK3C2B** NM_002646.3 | **RAF1** NM_002880.3 | **SPEN** NM_015001.2 | **WISP3** NM_198239.1 |
| **MYCN** NM_001293228.1 | **PIK3C2G** NM_001288772.1 | **RANBP2** NM_006267.4 | **SPOP** NM_001007226.1 | **WRN** NM_000553.4 |
| **MYD88** NM_001172567.1 | **PIK3C3** NM_002647.3 | **RARA** NM_000964.3 | **SPTA1** NM_003126.2 | **WT1** NM_024426.4 |
| **MYOD1** NM_002478.4 | **PIK3CA** NM_006218.3 | **RASA1** NM_002890.2 | **SRC** NM_198291.2 | **XIAP** NM_001167.3 |
| **NBN** NM_002485.4 | **PIK3CB** NM_006219.2 | **RB1** NM_000321.2 | **SRSF2** NM_003016.4 | **XPO1** NM_003400.3 |
| **NCOA3** NM_181659.2 | **PIK3CD** NM_005026.3 | **RBM10** NM_001204468.1 | **STAG2** NM_001042749.2 | **XRCC2** NM_005431.1 |
| **NCOR1** NM_006311.3 | **PIK3CG** NM_001282426.1 | **RECQL4** NM_004260.3 | **STAT3** NM_139276.2 | **XRCC3** NM_001100118.1 |
| **NEB** NM_001271208.1 | **PIK3R1** NM_181523.2 | **REL** NM_002908.3 | **STAT4** NM_001243835.1 | **YAP1** NM_001282101.1 |
| **NEGR1** NM_173808.2 | **PIK3R2** NM_005027.3 | **RET** NM_020975.4 | **STAT5A** NM_001288718.1 | **YES1** NM_005433.3 |
| **NF1** NM_000267.3 | **PIK3R3** NM_001303427.1 | **COP1** NM_022457.6 | **STAT5B** NM_012448.3 | **ZBTB2** NM_020861.2 |
| **NF2** NM_000268.3 | **PIM1** NM_001243186.1 | **RHEB** NM_005614.3 | **STK11** NM_000455.4 | **ZFHX3** NM_006885.3 |
| **NFE2L2** NM_006164.4 | **PLCG2** NM_002661.4 | **RHOA** NM_001664.3 | **STK40** NM_001282546.1 | **ZNF217** NM_006526.2 |
| **NFKBIA** NM_020529.2 | **PLK2** NM_006622.3 | **RICTOR** NM_001285439.1 | **SUFU** NM_016169.3 | **ZNF703** NM_025069.2 |
| **NKX2-1** NM_001079668.2 | **PMAIP1** NM_021127.2 | **RIT1** NM_001256821.1 | **SUZ12** NM_015355.3 | **ZNRF3** NM_001206998.1 |
| **NKX3-1** NM_006167.3 | **PMS1** NM_000534.4 | **RNF43** NM_017763.5 | **SYK** NM_001174167.2 | **ZRSR2** NM_005089.3 |
